# Complete Genome Sequence of Broad-Host-Range Shiga Toxin-Converting Bacteriophage SH2026Stx1, Isolated from Escherichia coli O157:H7

**DOI:** 10.1128/genomeA.00490-18

**Published:** 2018-06-21

**Authors:** Mingrui Duan, Samuel S. Hunter, Scott A. Minnich, Matthew W. Fagnan, Daniel D. New, Carolyn J. Hovde, Haiqing Sheng

**Affiliations:** a Bi-State School of Food Science, University of Idaho, Moscow, Idaho, USA; b Institute for Bioinformatics and Evolutionary Studies (IBEST), University of Idaho, Moscow, Idaho, USA

## Abstract

The Shiga toxin-encoding phage SH2026Stx1 was isolated from Escherichia coli O157:H7 strain 2026. SH2026Stx1 and its detoxified derivative can infect a broad range of E. coli strains, including commensal, enteropathogenic, and enteroaggregative strains. We report here the complete genome sequence of phage SH2026Stx1 and its important features.

## GENOME ANNOUNCEMENT

Shiga toxin (Stx)-producing Escherichia coli (STEC) strains are among the most important groups of foodborne pathogens due to their low infectious dose, causation of severe gastroenteritis, and propensity to cause hemorrhagic fever in infected pediatric and elderly individuals. Stx-converting phages, which belong to the lambdoid family of temperate bacteriophages (1), are responsible for the rise of STEC. They replicate either by a lytic cycle or as a prophage integrated into the bacterial genome. Since the first *stx-*associated disease report of E. coli O157:H7 in 1982, more than 250 STEC serogroups have been reported (2, 3). We isolated phage SH2026Stx1 after the induction of E. coli O157:H7 strain 2026 with mitomycin C. The phage produced clear plaques and has high titers on an indicator strain, E. coli K-12 (MG1655). To further study the phage, an *stx* deletion mutant was constructed. Subsequent testing showed that the Δ*stx-*derivative phage infected a wide variety of enteropathogenic E. coli, enteroaggregative E. coli, and commensal E. coli strains isolated from the stool microflora of healthy individuals. Because of its broad host range and small genome size, the Δ*stx* derivative phage may have application as a genetically engineered tool for the treatment of bacterial infections (4, 5). Here, we describe the complete genome sequence of SH2026Stx1.

The DNA of SH2026Stx1 was purified using a phage DNA isolation kit (Norgen Biotek Corp., Ontario, Canada). Phage DNA was sequenced using both Illumina MiSeq and MinION (Oxford Nanopore Technologies) sequencing platforms. For Illumina sequencing, a library was constructed by using an Illumina Nextera XL kit, followed by bead cleanup to remove small fragments. The quantitative PCR (qPCR)-quantified library was sequenced on a MiSeq instrument, using a v3 600-cycle kit to generate 2.5 million 300-bp paired-end reads. These reads were demultiplexed using bcl2fastq, and then PCR duplicates were removed and low-quality bases were trimmed using HTStream (https://github.com/ibest/HTStream ). For MinION sequencing, a phage DNA sample was prepared using the adapter ligation (SQK-LSK108) and native barcoding (EXP-NBD103) kits per manufacturer instructions and sequenced using an R9.4 Spot-On flow cell (FLO-MIN106). Samples were applied to a MinION Mk1B sequencer and run for 48 h. The resulting FAST5 files were base called and demultiplexed using Albacore v2.0.2. *De novo* assembly was done with Unicycler (6), utilizing the Illumina and Nanopore reads.

The genome sequence was initially annotated with the PHAge Search Tool Enhanced Release (PHASTER) (7) and refined by using Prokka v1.12 (8) with a custom database containing phage sequences from the Swiss-Prot database. This included six additional phage sequences (GenBank accession numbers NC_027984, NC_018846, NC_016158, NC_008464, NC_028656, and NC_011357) that were subjected to BLAST_HIT annotation by PHASTER.

The genome of SH2026Stx1 is 61,564 bp in length, with 78 protein-coding sequences and a GC content of 49.4%. The genomic structure of SH2026Stx1 resembles those of lambdoid phages and contained key regulatory components, such as N, Q, CI, CII, and CIII, a lysis cassette, and *stx*_1_ A and B genes. Interestingly, the SH2026Stx1 genome is more closely related to those of the Stx2-converting phages in the NCBI phage database (e.g., phages 933W [GenBank accession number NC_000924] and vB_EcoP-24B [GenBank accession number NC_027984] than to those of Stx1-converting phages.

### Accession number(s).

The SH2026Stx1 genome sequence was deposited in the GenBank database under accession number MG986485.

## References

[1] Schmidt H 2001 Shiga-toxin-converting bacteriophages. Res Microbiol 152:687–695. doi:10.1016/S0923-2508(01)01249-9. 11686382

[2] Johnson KE, Thorpe CM, Sears CL 2006 The emerging clinical importance of non-O157 Shiga toxin-producing *Escherichia coli*. Clin Infect Dis 43:1587–1595. doi:10.1086/509573. 17109294

[3] Sheng H, Davis MA, Knecht HJ, Hancock DD, Van Donkersgoed J, Hovde CJ 2005 Characterization of a Shiga toxin-, intimin-, and enterotoxin hemolysin-producing *Escherichia coli* ONT:H25 strain commonly isolated from healthy cattle. J Clin Microbiol 43:3213–3220. doi:10.1128/JCM.43.7.3213-3220.2005. 16000438 PMC1169089

[4] Yosef I, Manor M, Kiro R, Qimron U 2015 Temperate and lytic bacteriophages programmed to sensitize and kill antibiotic-resistant bacteria. Proc Natl Acad Sci U S A 112:7267–7272. doi:10.1073/pnas.1500107112. 26060300 PMC4466736

[5] Westwater C, Kasman LM, Schofield DA, Werner PA, Dolan JW, Schmidt MG, Norris JS 2003 Use of genetically engineered phage to deliver antimicrobial agents to bacteria: an alternative therapy for treatment of bacterial infections. Antimicrob Agents Chemother 47:1301–1307. doi:10.1128/AAC.47.4.1301-1307.2003. 12654662 PMC152521

[6] Wick RR, Judd LM, Gorrie CL, Holt KE 2017 Unicycler: resolving bacterial genome assemblies from short and long sequencing reads. PLoS Comput Biol 13:e1005595. doi:10.1371/journal.pcbi.1005595. 28594827 PMC5481147

[7] Arndt D, Grant JR, Marcu A, Sajed T, Pon A, Liang Y, Wishart DS 2016 PHASTER: a better, faster version of the PHAST phage search tool. Nucleic Acids Res 44:W16–W21. doi:10.1093/nar/gkw387. 27141966 PMC4987931

[8] Seemann T 2014 Prokka: rapid prokaryotic genome annotation. Bioinformatics 30:2068–2069. doi:10.1093/bioinformatics/btu153. 24642063

